# The effects of melatonin on bovine uniparental embryos development *in vitro* and the hormone secretion of COCs

**DOI:** 10.7717/peerj.3485

**Published:** 2017-07-07

**Authors:** Shujuan Wang, Baoru Liu, Wenju Liu, Yao Xiao, Hualin Zhang, Liguo Yang

**Affiliations:** 1 College of Animal Science, Anhui Science and Technology University, Bengbu, Anhui, China; 2 Key Lab of Agricultural Animal Genetics, Breeding and Reproduction of Ministry of Education, Huazhong Agriculture University, Wuhan, Hubei, China

**Keywords:** Melatonin, Bovine, Parthenogenetic embryo, Androgenetic embryo, Hormone, COCs, ROS

## Abstract

Melatonin is a unique multifunctional molecule that mediates reproductive functions in animals. In this study, we investigated the effects of melatonin on bovine parthenogenetic and androgenetic embryonic development, oocyte maturation, the reactive oxygen species (ROS) levels in parthenogenetic and androgenetic embryos and cumulus—oocyte complexes (COCs) hormone secretion with melatonin supplementation at four concentrations (0, 10, 20, and 30 pmol/mL), respectively. The results showed that melatonin significantly promoted the rates of bovine parthenogenetic and androgenetic embryonic cleavage and morula and blastocysts development (*P* < 0.05). The rate of cleavage was higher in the androgenetic embryo than that in the parthenogenetic embryo. Compared with the parthenogenetic embryos, the androgenetic embryos had a poor developmental competence from morula to blastocyst stage. Moreover, the levels of ROS were significantly lower in the parthenogenetic and androgenetic embryoes with melatonin-treated group than that of the control group (*P* < 0.05). Melatonin supplemented significantly increased the maturation rate of oocyte *in vitro* (*P* < 0.05). More importantly, melatonin significantly promoted the secretion of progesterone and estradiol by COCs (*P* < 0.05). To reveal the regulatory mechanism of melatonin on steroids synthesis, we found that steroidogenic genes (*CYP11A1, CYP19A1* and *StAR*) were upregulated, suggesting that melatonin regulated estradiol and progesterone secretion through mediating the expression of steroidogenic genes (*CYP11A1*, *CYP19A1* and *StAR*). In addition, MT1 and MT2 were identified in bovine early parthenogenetic and androgenetic embryos using western blot. It could be concluded that melatonin had beneficial effects on bovine oocyte *in vitro* maturation, COC hormone secretion, early development of subsequent parthenogenetic and androgenetic embryos. It is inferred that melatonin could be used to enhance the efficiency of *in vitro* developed embryos.

## Introduction

Despite great advances in assisted reproductive technology, the efficiency of *in vitro* developed embryos is still lower than *in vivo*-derived embryos. Previous studies showed significant differences in birth rates *in vivo* (76%) and *in vitro* (30%) (Abeydeera, 2002; Peterson & Lee, 2003). As for the uniparental embryos, the efficiency of developing to blastocyst is very low, especially the androgenetic embryo (Zhao et al., 2010; Xiao et al., 2013). Recently, the androgenetic embryos are produced through removing the maternal chromosomes, then injecting two spermatozoa into an oocyte (Vichera et al., 2011; Miki et al., 2009; Liang et al., 2009; Xiao et al., 2013; Zhang et al., 2014). Mammalian parthenogenetic embryos are obtained easily and efficiently, which have only oocyte-derived maternal genomes, and the androgenetic embryos that have only sperm-derived paternal genomes are produced difficultly (Sembon et al., 2012; Zhang et al., 2014). The early development of parthenogenetic and androgenetic embryos are limited, and arrest in the first 10 days and 8.5 days after gestation in mouse, respectively (Narasimha, Barton & Surani, 1997; Kono, 2006; Versieren et al., 2010). Moreover, our previous studies showed that the treatments of sperm capacitation and removal of the acrosome and plasma membrane prior to sperm injection and the histone deacetylase inhibitor were beneficial for early development of bovine androgenetic embryos, whereas the blastocyst formation rate was still low (Xiao et al., 2013; Zhang et al., 2014).

Considering the possible benefits of uniparental embryos, there is a great deal of studies about the mechanisms of the parthenogenetic and androgenetic embryos development. Uniparental embryos are an effective tool to explore genetic effects on the process of maternal and paternal genomic imprinting, as well as the contribution of the maternal and paternal genome in early embryonic development (Latham, Kutyna & Wang, 1999; Kono et al., 2004; Gebert et al., 2009; Sepulveda-Rincon et al., 2016; Ogawa et al., 2009). Some studies report that the addition of granulocyte colony-stimulating factor and valproic acid, AY9944A-7 and histone deacetylase inhibitor in the IVM medium improve the viability of parthenogenetic embryos in porcine (Cai et al., 2015; Huang et al., 2015), sheep (Hao et al., 2015) and androgenetic embryos in bovine (Zhang et al., 2014), respectively. Adding melatonin to the IVM medium could improve the development of parthenogenetic embryos in porcine (Kang et al., 2009). However, to the best of our knowledge, little is known about the effects of melatonin on bovine parthenogenetic and androgenetic embryos.

Melatonin (*N*-aceyl-5-methoxytryptamine) is a hormone rhythmically secreted from the pineal gland, but it is also produced from other source including portions of the ovary, the GCs and the oocyte (El-Raey et al., 2011; Sakaguchi et al., 2013; Acuña-Castroviejo et al., 2014; Reiter et al., 2014). In mammals, it plays an important role in the reproductive system (Reiter, 1991), circadian rhyhms (Dubocovich et al., 1998), antioxidant activity (Tamura et al., 2012) via binding to the melatonin receptors (MT1 and MT2). Melatonin as a direct radical scavenger and indirect antioxidant play an important role in protecting ovarian tissues from oxidative stress (Tamura et al., 2012; Manchester et al., 2015; Reiter et al., 2016). *In vitro* handling and culture expose oocytes and embryos to oxidative stress resulting from various environmental factors (Guerin, EI & Menezo, 2001), while ROS induce cell membrane and DNA damage and play a role in apoptosis (Kang et al., 2009). A high level of ROS blocking *in vitro* embryonic development and oocyte maturation have been reported (Guerin, EI & Menezo, 2001; Matsuzuka et al., 2005; Juknat et al., 2005; Wang et al., 2012b). Recent studies mainly focus on the effects of melatonin on oocyte maturation and embryo development via inhibiting ROS formation (Wang et al., 2014; Kang et al., 2009; Choi et al., 2008; Tian et al., 2014). On the other hand, melatonin regulates the expression of superoxide dismutase and glutathione peroxidase to scavenge the ROS (Tatemoto et al., 2004; Wang et al., 2014). The concentration of melatonin in the follicular fluid is threefold higher than that in peripheral blood serum concentration (Yie et al., 1995); it is possible that melatonin is the most effective antioxidant in the follicle, and directly protects the oocytes from ROS (Tatemoto et al., 2004; Fatehi et al., 2005; Tamura et al., 2012). Therefore, it is an effective way that reduces the ROS level to improve *in vitro* oocytes maturation and embryos development against oxidative stress.

Melatonin has been well known as a free radical scavenger and antioxidant, as well as an important anti-apoptotic agent, which have drawn increased attention on oocyte and embryonic development. More importantly, the recent studies indicate that melatonin promotes the embryonic development in mouse, sheep, pig and cattle (Abecia, Forcada & Zúñiga, 2002; Kang et al., 2009; Tian, Wen & Shi, 2010; Gao et al., 2012; Wang et al., 2014). In addition, melatonin improves blastocyst formation rate, mean cell number/blastocyst, and the rate of hatch ability in the 2-cell mouse embryos (Tian, Wen & Shi, 2010). Similar observations indicate that melatonin enhances the maturation of oocytes, blastocyst rate, mean cell number/blastocyst, and the rate of hatch blastocyst *in vitro* fertilization bovine embryo development (Wang et al., 2014). Moreover, the maturation rate of oocytes, parthenogenetic activation rate of blastocyst and cell numbers of blastocyst are improved after melatonin treatment by decreasing the ROS in the porcine embryos (Shi et al., 2009; Kang et al., 2009). Melatonin promotes *in vitro* development of pronuclear embryos and increases the efficiency of blastocyst implantation in murine (Wang et al., 2013). Based on the beneficial effects of melatonin in scavenging ROS, and modulating antioxidant and anti-apoptosis, the potential mechanisms of melatonin on embryo development may rely on its antioxidative and anti-apoptotic capacities (Wang et al., 2014; Gao et al., 2012).

Androgenetic embryos contain only sperm-derived paternal genomes, through removal of the maternal chromosomes, followed by injection of two spermatozoa into an oocyte. Parthenogenetic embryos are produced through incorporating oocyte-derived maternal genomes. Therefore, the androgenetic and parthenogenetic embryos are a valuable resource for providing an insight into the epigenetic reprogramming events and investigating the contribution of the paternal or maternal genome to early embryonic development during gametogenesis and embryogenesis (Obata et al., 2000; Ogawa et al., 2009; Xiao et al., 2014; Zhang et al., 2014). Moreover, parthenogenetic and androgenetic embryonic stem cells derived from the inner cell mass of blastocysts are histocompatible and offer an important autologous source of pluripotent stem cells that can be used for therapeutical transplantation (Zhao et al., 2010). However, the parthenogenetic and androgenetic blastocyst formation rate is very low. Taking into account the importance of melatonin in anti-apoptosis, scavenging the ROS and regulating the reproduction, it suggests that melatonin might have precise effects on bovine oocyte maturation and parthenogenetic and androgenetic embryo development. Therefore, the present study is designed with the following objectives: (i) to evaluate the effects of melatonin on parthenogenetic and androgenetic embryo development, (ii) to measure the effects of melatonin on secretion of progesterone and estradiol by COCs, and (iii) to identify the expression of MT1 and MT2 in early parthenogenetic and androgenetic embryo.

## Materials and Methods

### *In vitro* maturation of oocyte

Oocyte collection and *in vitro* maturation (IVM) were performed as our previously described (Gao et al., 2007; Xiao et al., 2013) with little modifications. Briefly, bovine ovaries were obtained from the Wuhan slaughterhouse and immediately transported to the laboratory in sterile Dulbecco’s phosphate-buffered saline (D-PBS) containing penicillin (100 IU/mL), streptomycin (100 µg/mL) and mycostatin (50 IU/mL) at 28−30 °C within 3 h. Opaque or hemorrhagic follicles were discarded. COCs were aspirated from the follicles (3–6 mm in diameter) with an 18-gauge needle attached to a disposable 10-mL syringe. COCs with intact compact cumulus layers were selected and washed three times in the maturation medium. Each group of 30–40 COCs was transferred to 500 µL of tissue culture medium-199 (catalog no. 11150-059; Gibco, Gaithersburg, MD, USA) supplemented with 10% fetal bovine serum (catalog no. 10099-141; Gibco, Gaithersburg, MD, USA), 2.5 mM sodium pyruvate, 1 ng mL^−1^ estradiol-17β, 0.5 IU mL^−1^ FSH (Chinese Academy of Science, Beijing, China), 0.5 IU mL^−1^ LH (Chinese Academy of Science, Beijing, China), 50 ng mL^−1^ epidermal growth factor (Invitrogen, CA, USA), and 1% penicillin-streptomycin (Pen-Strep; 10,000 IU mL^−1^ and 10,000 µg mL^−1^, respectively; Gibco, Gaithersburg, MD, USA). The medium was covered with sterile mineral oil in a four-well dish. Oocytes were matured at 38.5 °C in an atmosphere of 5% CO_2_ for 22–24 h. After maturation, the cumulus cells of COCs were removed by vortexing for 5 min in 0.1% bovine testicular hyaluronidase in 10mM HEPES-buffered Tyrode’s albumin lactate pyruvate medium (HEPES-TALP) (Parrish et al., 1988; Xiao et al., 2013). Oocyte maturation was determined by evaluating the presence of polar body. The denuded oocytes were fixed in methanol for 15 min and stained with Hoechst 33342 (Sigma-Aldrich Corp, St. Louis, MO, USA) in PBS. After 22–24 h IVM, following staining procedure, oocytes were mounted on a glass slide and evaluated under a phase-contrast microscope (Nikon, Tokyo, Japan). In this study, the protocols for the experiment was reviewed and approved by the Institutional Committee on Animal Care and Use at Huazhong Agricultural University.

### RNA extraction and Real-Time PCR

The COCs total RNA were isolated using RNAprep pure cell Kit (Tiangen, Beijing China), After 22–24 h IVM. To remove the genomic DNA, the total RNA was treated with RNase- free DNaseI. The total RNA were reverse-transcribed to cDNA with a RevertAid First Strand cDNA Synthesis Kit (Thermo Scientific, Waltham, MA, USA) according to the manufac-turer’s instructions. The quantitative real-time PCR was performed using LightCycler 480 II Real-Time PCR System (Roche, Basel, Switzerland). The amplification reaction was carried out with LightCycler 480 SYBR Green I Master. The specific primer pairs were listed in Table 1. The expression levels of the target genes were normalized to *β-actin* in each sample. The related mRNA expression levels were estimated using the formula: 2^−△△*CT*^ (Livak & Schmittgen, 2001).

**Table 1:** Sequences of primer pairs for quantitative real-time PCR.

Gene		Forward primer sequence (5′ → 3′)	Reverse primer sequence (5′ → 3′)	Length
*CYP11A1*	ATGCTGGAGGAGACAGTGAACC		GCAGTAGAGGATGCCTGGGTAA	249
*CYP19A1*	CACCCATCTTTGCCAGGTAGTC		ACCCACAGGAGGTAAGCCTATAAA	78
*StAR*	GTG GAT TTT GCC AAT CAC CT		TTATTG AAA ACG TGC CAC CA	203
*β-actin*	CATCGGCAATGAGCGGTTCC		CCGTGTTGGCGTAGAGGTCC	145

### Radioimmunoassay (RIA) for progesterone and estradiol *in vitro* maturation medium of oocytes

*In vitro* maturation medium of bovine oocytes was collected and frozen at −20 °C. Concentrations of progesterone and estradiol were measured by iodine [^125^I] progesterone and estradiol radioimmunoassay kits, respectively (Chemclin Biotech, Co., Beijing, China). The assays had sensitivity as follows: progesterone, ≤0.05 ng mL^−1^ and estradiol, ≤3 pg mL^−1^. The antibodies used in the kits had no cross-reactivity with melatonin.

### Activation of matured oocytes and *in vitro* culture of activated parthenogenetic embryos

After 22–24 h of IVM, the oocytes were activated with 5 µM ionomycin for 5 min, cultured in the IVM medium for 2 h at 38.5 °C under 5% CO_2_ in humidified air, and subsequently treated with 2mM 6-dimethylaminopurine for 4 h under the same conditions. The activated oocytes were washed three times with a synthetic oviduct fluid (SOF) medium supplemented with amino acids and bovine serum albumin (BSA) (SOFaa), and placed in an embryo culture medium (SOFaa). Parthenogenetically activated oocytes (15–20 cells well^−1^) were cultured in 100 µL droplets of SOFaa and finally placed at 38.5 °C under 5% CO_2_ in humidified air. The cleavage and blastocyst development rates were observed and recorded at 48 and 168 h after *in vitro* culture, respectively.

### Sperm preparation

Sperm preparation was carried out as our previously described (Xiao et al., 2013; Zhang et al., 2014). Briefly, commercially available frozen bull semen (Beijing Dairy Cattle Center, Beijing, China) was thawed in a water bath at 37.5 °C for 15 s. The thawed semen was overlayered on a Percoll density gradient consisting of 45% and 90% Percoll in a 15 mL conical tube. The tube was centrifuged for 10 min at 600 g, and the sperm pellet was resuspended in modified sperm-Tyrode’s albumin lactate pyruvate (Sp-TALP) (Parrish et al., 1988) without calcium and BSA in 1.5-mL tubes, washed, and centrifuged twice for 5 min at 600 g each. The spermatozoa were then incubated in a capacitating medium for 4 h at 38.5 °C in an atmosphere of 5% CO_2_. Following incubation, the spermatozoa were incubated in Sp-TALP supplemented with 0.1 mg mL^−1^ lysolecithin for 30 min to remove the acrosome and plasma membrane. For the removal of lysolecithin, the spermatozoa were washed twice by centrifugation for 10 min at 600 g in 1 mL of Sp-TALP. The sperm pellet was resuspended in HEPES-TALP and kept at 38.5 °C until transferred into micromanipulation drops.

### Reconstruction of androgenetic embryos

Reconstruction of androgenetic embryos was carried out in accordance with the methods described in our previous studies (Xiao et al., 2013; Zhang et al., 2014). Briefly, micromanipulation was performed at 200× magnification with a micromanipulation system (Narishige, Tokyo, Japan) attached to an inverted microscope (TE2000 U; Nikon, Tokyo, Japan). Approximately 20 denuded oocytes with a visible first polar body and uniform ooplasm were selected and stained with 10 mg mL^−1^ Hoechst 33342 for 10 min before being enucleated by aspiration of the polar body and metaphase II (MII) plate with the assistance of a brief (<10 s) exposure to ultraviolet light. Just before injection of sperms, micromanipulation droplets were prepared with 30 µL HEPES-TALP medium containing 5 mg mL^−1^ cytochalasin B and 10 µL polyvinylpyrrolidone. The sperm suspension were placed into a 60-mm culture dish and covered with mineral oil. Selected spermatozoa were aspirated tail first into the injection pipette, and two sperm cells were injected into an enucleated oocyte through the same hole of the zona pellucida. After injection, the oocytes were immediately transferred to SOF culture droplets and cultured for 2 h in a CO_2_ incubator. Reconstructed oocytes were exposed to 5 mM ionomycin calcium salt for 5 min and cultured in the SOF medium with 1.9 mM 6-dimethylaminopurine for 4 h at 38.5 °C in a humidified atmosphere of 5% CO_2_ in air. Droplets of 500 µL of SOF containing 3 mg mL^−1^ BSA were prepared in a four-well dish under mineral oil and equilibrated for 2 h before loading of embryos (30–40 embryos well^−1^). Activated oocytes were washed three times with SOF containing 3 mg mL^−1^ BSA and transferred to the four-well dish as prepared earlier for further culture or treated with melatonin as described later. Embryonic development was observed at 48 and 168 h of culture under an inverted microscope (TE2000 U; Nikon, Tokyo, Japan).

### Measurement of ROS levels in embryos

The levels of ROS in parthenogenetic and androgenetic embryos was were examined according to the dichlorohydrofluorescein diacetate (DCHFDA, Sigma, USA) method described by Yang et al. (1998) and Hashimoto et al. (2000) with minor modifications. After 48 h of *in vitro* culture, the embryos were transferred to the culture medium, incubated in a culture medium containing 10 mM DCHFDA, and washed three times in a new IVM medium after 15 min. The fluorescent emissions from the embryos were recorded as tagged image file format files using a cooled charge-coupled device camera attached to a fluorescence microscope (TS100; Nikon, Tokyo, Japan) with excitation filters at 405–435 and 515 nm for emission. The recorded fluorescent images were analyzed using Image-Pro Plus 6.0 (Media Cybernetics Co, Rockville, MD, USA) by mean gray values of fluorescence. The experiment was replicated three times with 25–30 embryos in each replicate.

### Expression of MT1 and MT2 in embryos

Bovine parthenogenetic and androgenetic embryos at the morula stage were washed three times with polyvinyl alcohol-D-PBS, lysed in sodium dodecyl sulfate polyacrylamide gel electrophoresis loading buffer, heated to 100 °C for 5 min, and frozen at −80 °C until use. The proteins were separated using a 12% polyacrylamide gel and then transferred to apolyvinylidene fluoride (PVDF) membrane for 1 h under a 200-mA electric current. The PVDF membranes were blocked by 5% nonfat milk at 4 °C overnight, incubated with primary antibodies (MEL-1A-R sc-13186, MEL-1B-R sc-13177; Santa Cruz Biotechnology Inc, USA; 1:400 dilution) at 4 °C overnight, washed, and further incubated with HRP-goat anti-rabbit IgG (sc-2004; 1:5,000 dilution; Santa Cruz Biotechnology, Santa Cruz, CA, USA) for 1 h at 37 °C. Immunoreactive bands were detected using the Bio-Rad Clarity Western ECL kit (Bio-Rad, Hercules, CA, USA) and scanned using a chemiluminescent imaging system (Bio-Rad ChemiDoc XRS, Hercules, CA, USA). The PVDF membranes were then stripped and reprobed with mouse monoclonal antibody (β-actin sc47778; 1:1,000 dilution; Santa Cruz Biotechnology, Santa Cruz, CA, USA) for normalization.

### Statistical analysis

Unless stated otherwise, data were presented as the mean ± s.e.m. All the experiments were repeated at least thrice. Multiple group comparisons were performed by one-way analysis of variance with the Tukey’s honest significant difference post-hoc test using SAS 9.0 system software (SAS Institute Inc., Cary, NC, USA). A value of *P* less than 0.05 was considered to be significant.

## Results

### Effects of melatonin on *in vitro* maturation (IVM) of bovine oocytes

A total of 1,049 oocytes were used in eleven replicates to evaluate the effects of melatonin on oocyte maturation. The maturation rate of oocyte was significantly higher in the melatonin supplemented groups than that in the control group (*P* < 0.05). In the melatonin-treated groups, the effect of 30 pmol mL^−1^ melatonin (67.40 ± 3.80) on oocyte maturation rate was significantly higher than that of 10 pmol mL^−1^ melatonin (51.32 ± 7.32), 20 pmol mL^−1^ melatonin (57.47 ± 5.65) and control group (41.60 ± 14.31) (Table 2) (*P* < 0.05). Also, the rate of oocyte maturation was increased in the 20 pmol mL^−1^ melatonin group when compared with that in the 10 pmol mL^−1^ melatonin group, but there was no significant difference (*P* > 0.05).

**Table 2:** Effects of melatonin on oocytes *in vitro* maturation.

Melatonin (pmol∕ml)	COCs (n)	Maturation rate of oocytes (% ± s.e.m)
0	251	41.60 ± 14.31^a^
10	285	51.32 ± 7.32^b^
20	260	57.47 ± 5.65^b^
30	253	67.40 ± 3.80^c^

**Notes.**

The data with different letters indicates the level of significance in column (*P* < 0.05).

### Effect of melatonin on ROS in bovine embryos

**Figure 1:**
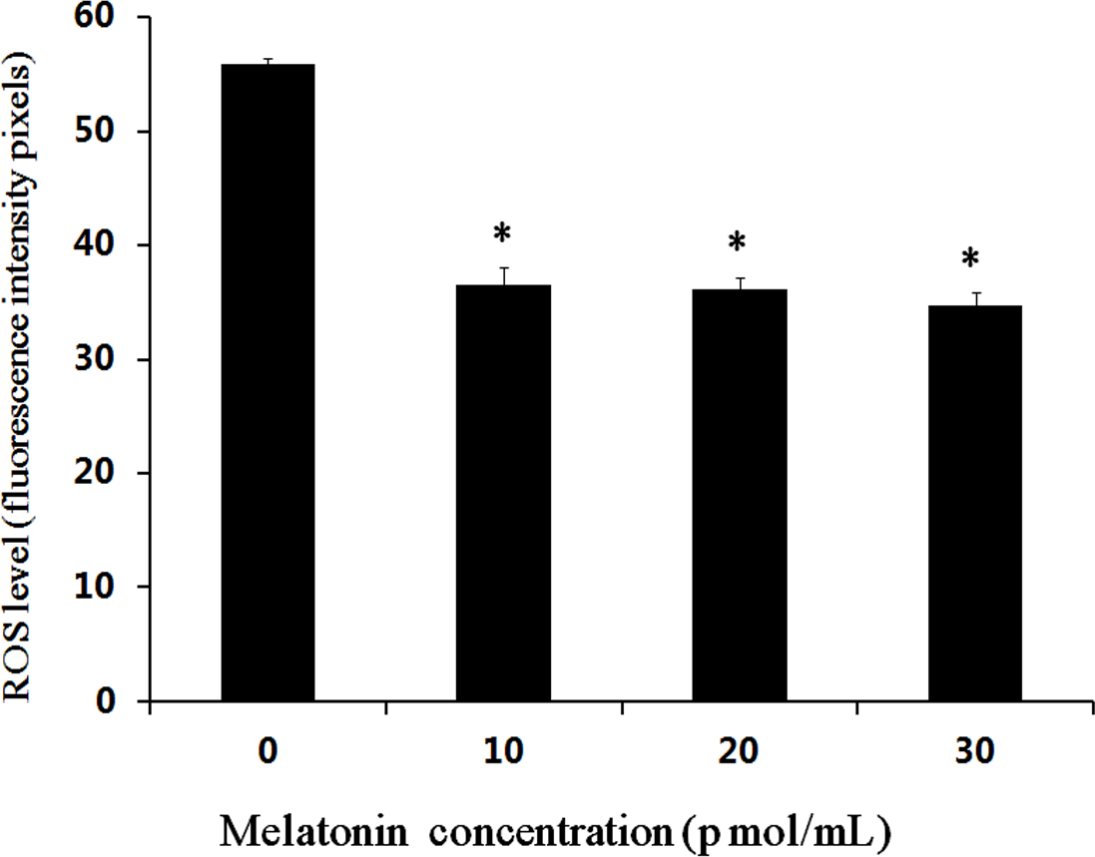
Effects of melatonin on ROS levels in bovine cultured parthenogenetic embryos. The * indicates the level of significance (*P* < 0.05).

**Figure 2:**
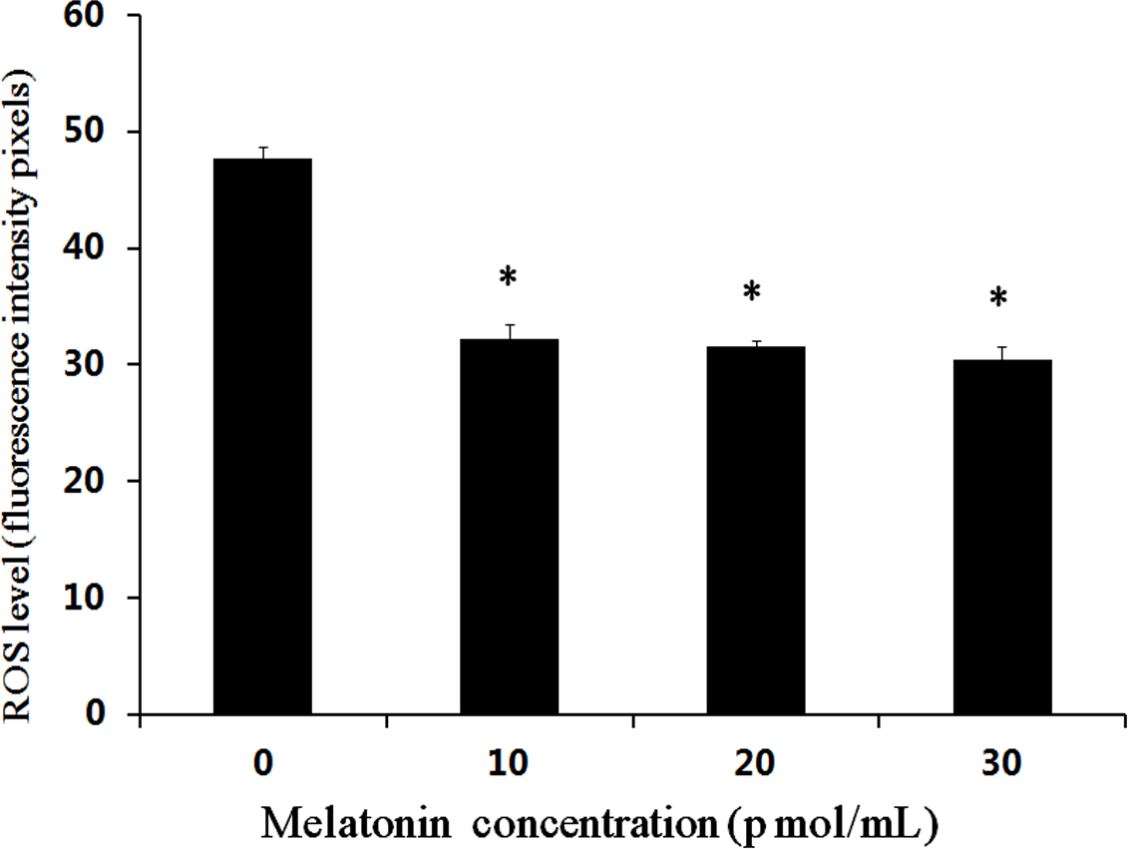
Effects of melatonin on ROS levels in bovine cultured androgenetic embryos. The * indicates the level of significance (*P* < 0.05).

A total of 432 parthenogenetic and androgenetic embryos were used to evaluate the effect of melatonin on the levels of ROS using DCHF-DA staining. The results showed that the mean fluorescence intensity pixel with melatonin supplemented groups were significantly lower than that in the control group (*P* < 0.05) (Figs. 1 and 2). However, no significant difference was observed among the groups supplemented with different concentrations of melatonin.

### Effect of melatonin on hormone secretion of COCs

The oocyte *in vitro* maturation medium supplemented with melatonin was collected and frozen at −20 °C at 22–24 h. The concentration of progesterone and estradiol were measured by radioimmunoassay method (Table 3). Melatonin significantly promoted the secretion of progesterone by COCs with melatonin-treated (*P* < 0.05). The estradiol production in COCs was higher in the melatonin-treated group than that in control groups (*P* < 0.05), with no significant difference between 10 pmol mL^−1^ and control group (*P* > 0.05). Moreover, melatonin had dose-dependent effects on promoting the secretion of progesterone and estradiol.

**Figure 3:**
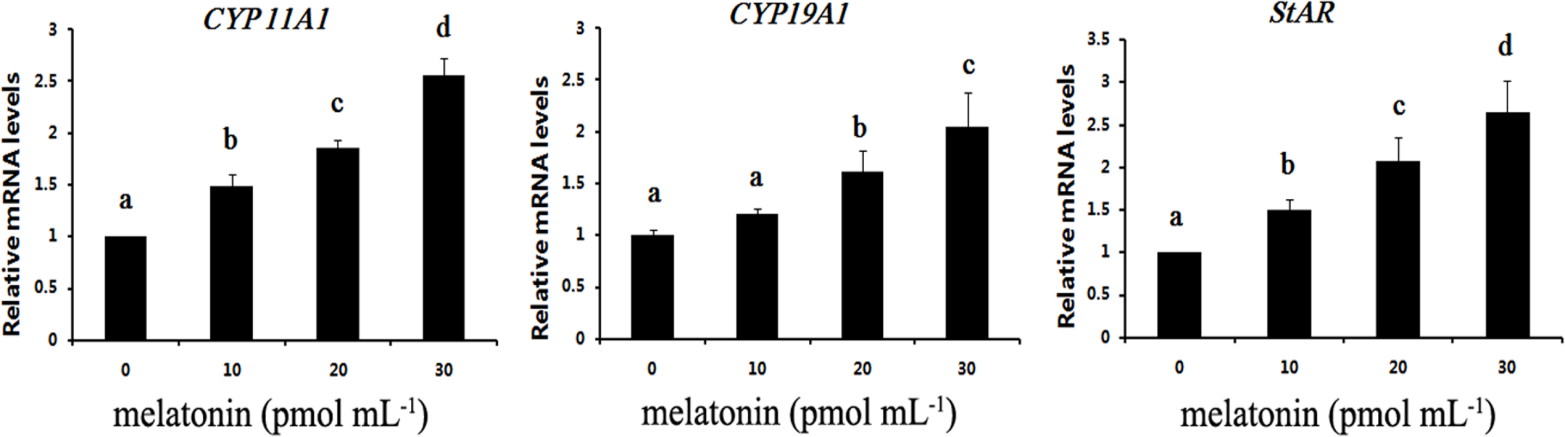
Effects of melatonin supplementation on expression of *CYP 11A1*, *CYP19A1* and *StAR* in COCs. The mRNA levels of *CYP 11A1*, *CYP19A1* and *StAR* were examined by real-time PCR. The data with different letters (a, b, c and d) were significant difference (*P* < 0.05).

To further elucidate the effect of melatonin on hormone secretion by COCs, the expression of steroidogenic genes (*CYP 11A1*, *CYP19A1* and *StAR*) were determined by real-time PCR. Coinciding with the effect of melatonin on the secretion of progesterone and estradiol, the present results indicated that melatonin significantly upregulated the expression of *CYP 11A1*, *CYP19A1* and *StAR* (*P* < 0.05, Fig. 3), and there was no significant difference in the expression of *CYP19A1* between 10 pmol mL^−1^ and control group (*P* > 0.05). Moreover, melatonin had dose-dependent effects on upregulating the expression of *CYP 11A1*, *CYP19A1* and *StAR* and the expression of *CYP 11A1*, *CYP19A1* and *StAR* was higher in the 30 pmol mL^−1^ melatonin-treated group. Therefore, melatonin mediate estradiol and progesterone secretion through regulating the expression of steroidogenic genes (*CYP11A1*, *CYP19A1* and *StAR*).

**Table 3:** The hormone concentrations in different melatonin concentrations maturation medium.

MT (pmol∕ml)	P (ng/mL)	E_2_ (pg/ml)
0	0.06 ± 0.01^a^	1575.24 ± 4.55^a^
10	0.15 ± 0.02^b^	1589.44 ± 5.70^a^
20	0.22 ± 0.02^c^	1618.36 ± 3.39^b^
30	0.35 ± 0.02^d^	1679.62 ± 6.34^c^

**Notes.**

The data with different letters indicates the level of significance in column (*P* < 0.05).

### Effect of melatonin on the development of bovine parthenogenetic embryos

**Figure 4:**
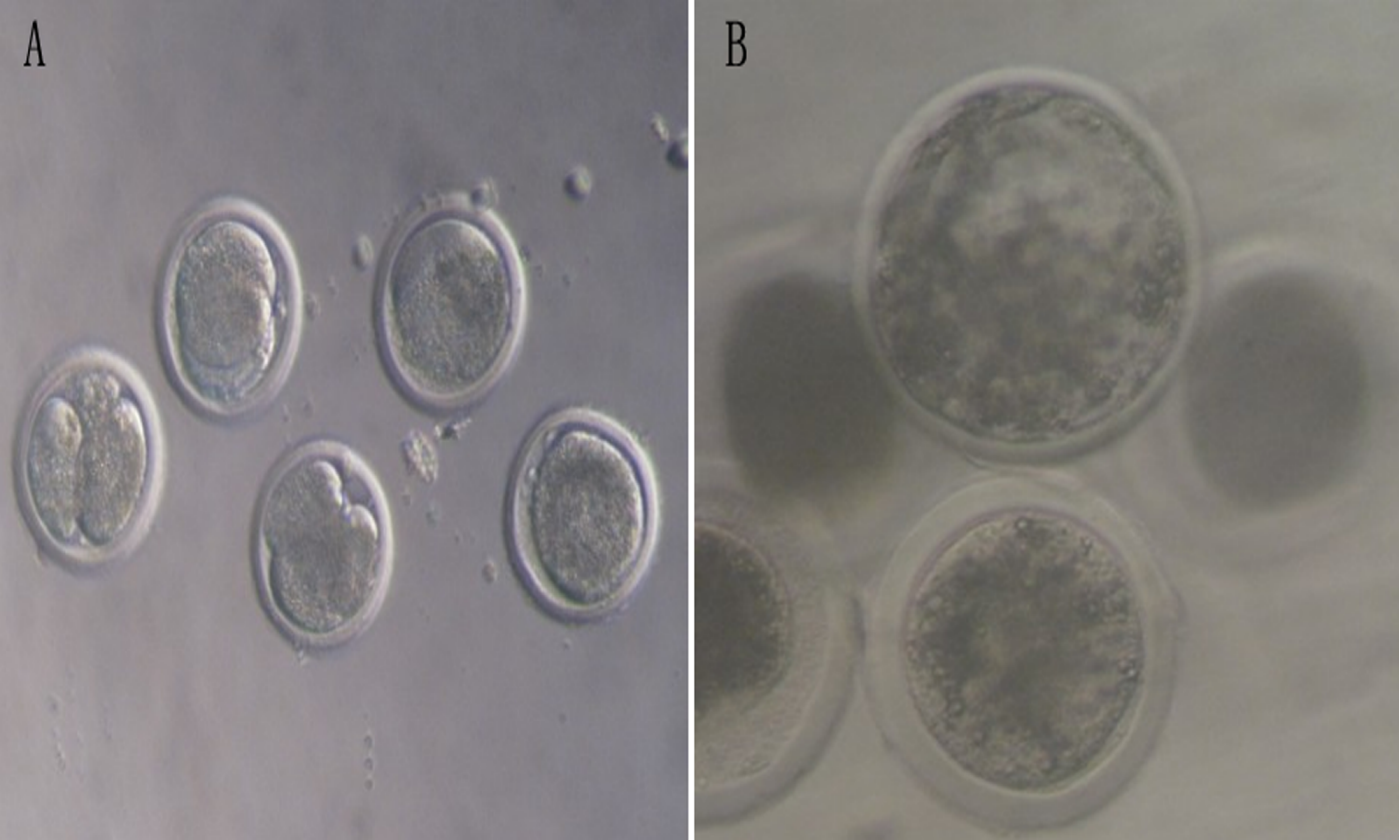
The development of bovine parthenogenetic embryos (×200). (A) Bovine parthenogenetic embryos at cleavage stage; (B) Bovine parthenogenetic embryos at blastocyst stage.

A total of 567 oocytes were used to determine the effects of different concentrations of melatonin on the parthenogenetic embryo development (Table 4, Fig. 4). Three concentrations of melatonin (10 pmol mL^−1^, 20 pmol mL^−1^ and 30 pmol mL^−1^) were added into SOFaa for seven days. The results showed that the rates of cleavage and morula were significantly higher in the 10, 20, and 30 pmol mL^−1^ melatonin-treated groups (67.25 ± 13.35 and 48.21 ± 10.65; 69.03 ± 9.85 and 51.33 ± 20.22; 66.76 ± 11.54 and 52.96 ± 10.73, respectively) than that in the control group (44.44 ± 6.77 and 30.86 ± 6.63, respectively; *P* < 0.05), and there were no significant difference among the melatonin-treated groups (Table 4). The rates of blastocyst was significntly increased in the 30 pmol mL^−1^ melatonin-treated group (22.02 ± 4.44) than that of 10, 20 melatonin-treated groups (19.55 ± 3.55 and 20.01 ± 9.57, respectively) and control group (14.43 ± 7.48) (*P* < 0.05), and there were no significant difference among 10, 20 melatonin-treated and control groups. However, The rates of blastocyst showed a tendency to increased in the melatonin-treated groups.

**Table 4:** Effect of melatonin on the development of bovine parthenogenetic embryos.

Melatonin (pmol/mL)	Parthenogenetic embryos (n)	Rate of cleavage (*%*±*s.e.m* )	Rate of morula (*%*±*s.e.m*)	Rate of blastocyst (*%*±*s.e.m*)
0	133	44.44 ± 6.77^a^ (59/133)	30.86 ± 6.63^a^ (41/133)	14.43 ± 7.48^a^ (9/59)
10	140	67.25 ± 13.35^b^ (95/140)	48.21 ± 10.65^b^ (68/140)	19.55 ± 3.55^a^ (19/95)
20	149	69.03 ± 9.85^b^ (103/149)	51.33 ± 20.22^b^ (45/149)	20.01 ± 9.57^a^ (21/103)
30	145	66.76 ± 11.54^b^ (96/145)	52.96 ± 10.73^b^ (46/145)	22.02 ± 4.44^b^ (21/96)

**Notes.**

The data with different letters indicates the level of significance in column (*P* < 0.05).

### Effect of melatonin on the development of bovine androgenetic embryos

**Figure 5:**
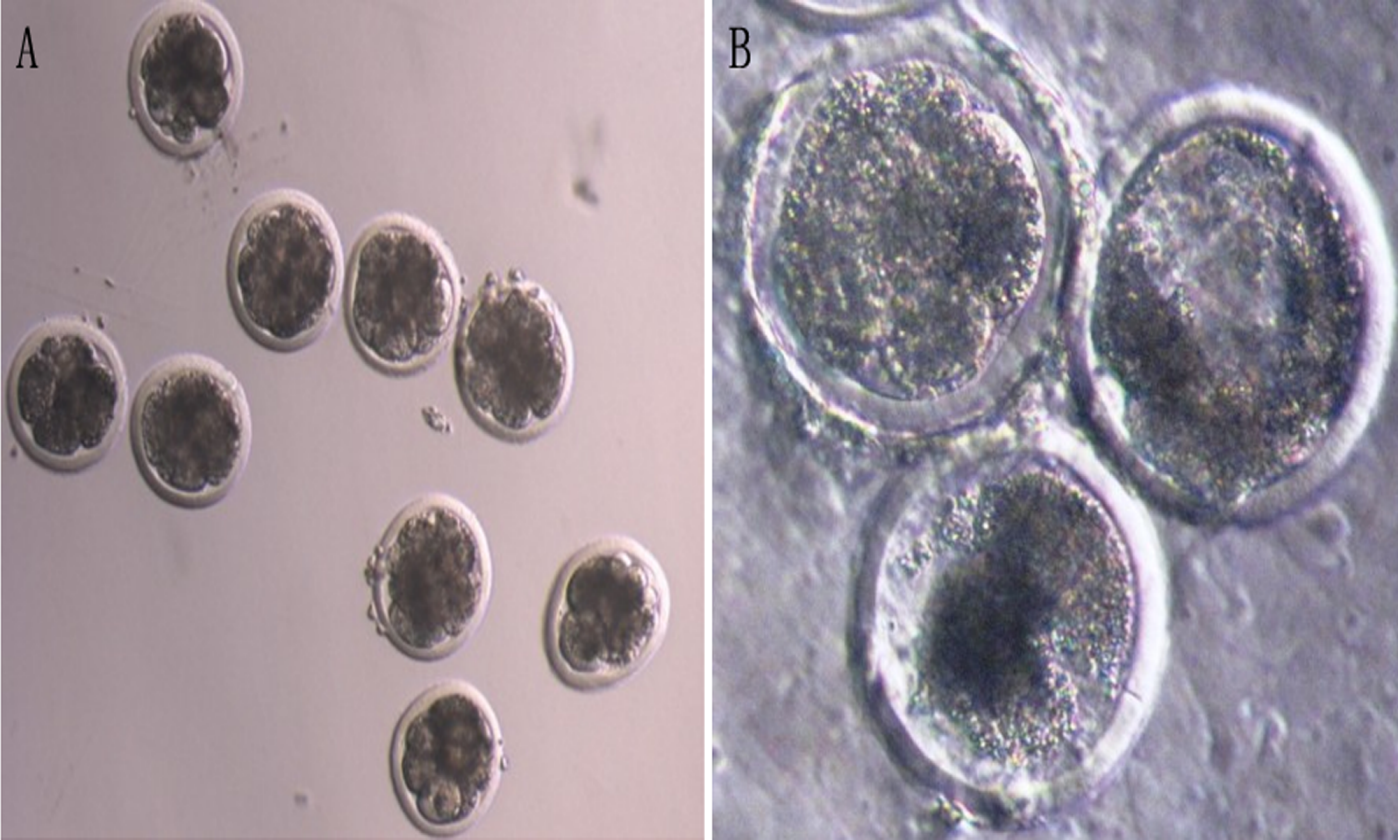
The development of bovine androgenetic embryos (×200). (A) Bovine androgenetic embryos at cleavage stage; (B) bovine androgenetic embryos at blastocyst stage.

A total of 412 androgenetic embryos were used to determine the effects of melatonin on the androgenetic embryos development (Table 5, Fig. 5). The results showed that the rates of cleavage in the 20 pmol mL^−1^ (76.38 ± 3.90) and 30 pmol mL^−1^(77.25 ± 4.28) melatonin-treated groups were significantly higher than those in the 10 pmol mL^−1^ (70.14 ± 4.34) and the control group (55.46 ± 9.03) (*P* < 0.05). Moreover, the rates of cleavage in 20 pmol mL^−1^ and 30 pmol mL^−1^ melatonin-treated groups, and the 10 pmol mL^−1^ and the control group did not show a significant difference (*P* > 0.05), respectively. The rates of morula and blastocyst in the 10 pmol mL^−1^ (35.42 ± 5.51 and 13.10 ± 4.44, respectively), 20 pmol mL^−1^ (38.26 ± 2.13 and 14.26 ± 3.13, respectively), and 30 pmol mL^−1^ (37.58 ± 1.42 and 14.06 ± 1.46, respectively) melatonin-treated groups were significantly higher than those in the control group (19.22 ± 1.36 and 0, respectively; *P* < 0.05). However, there were no significant differences among the melatonin-treated groups (*P* > 0.05).

**Table 5:** Effect of melatonin on the development of bovine androgenetic embryos.

Melatonin (pmol/mL)	Androgenetic embryos (n)	Rate of cleavage (% ± S.E.M )	Rate of morula (% ± S.E.M)	Rate of blastocyst (% ± S.E.M)
0	104	55.46 ± 9.03^a^ (58∕104)	19.22 ± 1.36^a^ (20∕104)	0^a^ (0∕58)
10	86	70.14 ± 4.34^a^ (60∕86)	35.42 ± 5.51^b^ (30∕86)	13.10 ± 4.44^b^ (8∕60)
20	110	76.38 ± 3.90^bc^ (84∕110)	38.26 ± 2.13^b^ (42∕110)	14.26 ± 3.13^b^ (12∕84)
30	112	77.25 ± 4.28^c^ (86∕112)	37.58 ± 1.42^b^ (42∕112)	14.06 ± 1.46^b^ (12∕86)

**Notes.**

The data with different letters indicates the level of significance in column (*P* < 0.05).

### Expression of melatonin receptors in bovine parthenogenetic and androgenetic embryos

**Figure 6:**
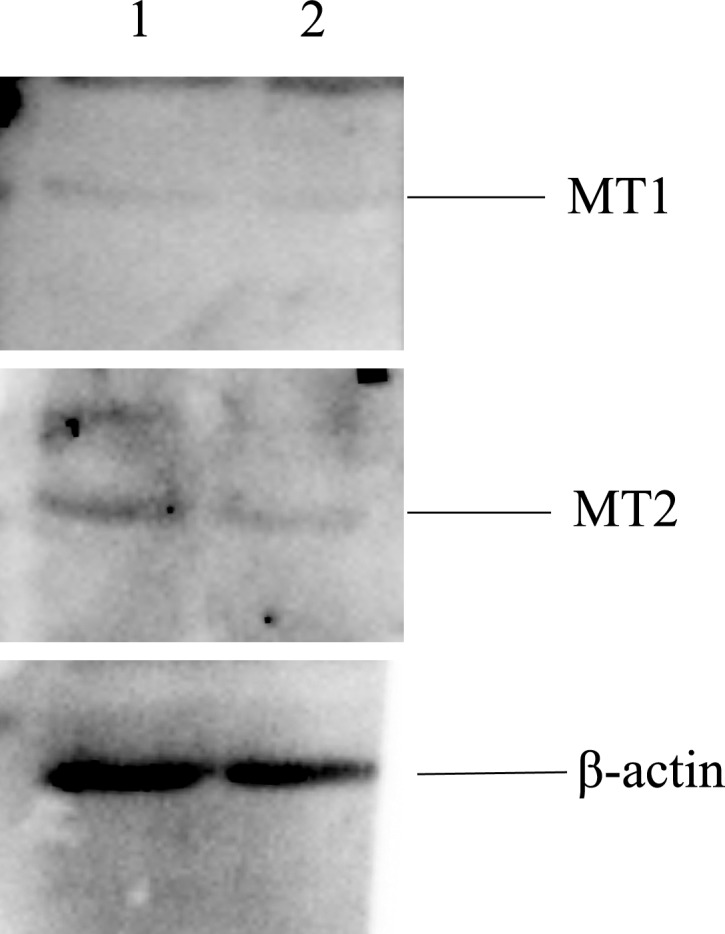
Proteins of MT1 and MT2 in the morula stage of parthenogenetic and androgenetic embryos detected by the Western blotting method. 1: parthenogenetic embryos; 2: androgenetic embryos.

The expressions of melatonin receptors (MT1 and MT2) in bovine parthenogenetic and androgenetic embryos at the morula stage were examined using western blot. The results revealed the presence of MT1 and MT2 in early bovine parthenogenetic and androgenetic embryos (Fig. 6).

## Discussion

Parthenogenetic and androgenetic embryos are a potential resource for embryonic stem cells, cell therapy and serve as a model for studying the mechanisms of maternal or paternal genomic imprinting. However, the poor developmental ability of uniparental embryos has limited the use of the most promising source. Numerous researches have indicated the failure of embryo development to somite stages in mouse androgenetic embryos (McGrath & Solter, 1984), term in mouse parthenogenetic embryos (Surani, Barton & Hum, 1983), day 9.5 of gestation in mouse androgenetic and parthenogenetic embryos (Obata et al., 2000; Ogawa et al., 2009) and blastocyst stages in bovine (Xiao et al., 2013; Zhang et al., 2014). Many factors are involved in the development deficiency of parthenogenetic and androgenetic embryos, such as defects of trophoblast stem cells (Ogawa et al., 2009), inappropriate expression of maternal and paternal imprinted genes (Surani, Barton & Norris, 1984; McGrath & Solter, 1984; Vichera et al., 2011; Ogawa et al., 2006; Hu et al., 2015), insufficient sperm decondensation and asynchronous pronucleus formation (Lagutina et al., 2004; Xiao et al., 2013), and the poor extra embryonic growth (Ogawa et al., 2009). Our previous studies have successfully constructed the bovine androgenetic embryos, and the efficiency of development to blastocysts is enhanced by sperm chromatin remodeling and epigenetic regulation of the sperm chromatin, however, the efficiency is still low (Xiao et al., 2013; Zhang et al., 2014). In the present study, we presented results that melatonin improved the development of bovine parthenogenetic and androgenetic embryos. In addition, melatonin promoted oocytes *in vitro* maturation and the secretion of progesterone and estradiol in COCs. One encouragement is that the results of similar studies that melatonin improves the *in vitro* maturation rate of oocytes and the development of embryos in mice (Ishizuka et al., 2000), cattle (El-Raey et al., 2011) and pig (Choi et al., 2008; Kang et al., 2009). Melatonin is involved in metabolism, formation of blastocysts, and embryonic development at certain levels (Jang et al., 2005). The present study found that melatonin improved the cleavage rate and the rate of morula and blastocyst development in bovine parthenogenetic and androgenetic embryos treated with 10, 20, and 30 pmol mL-1 melatonin. Similar observations indicate that melatonin enhances the maturation of oocytes and blastocyst rate *in vitro* fertilization bovine embryo development and improves blastocyst rate in the 2-cell mouse embryos (Tian, Wen & Shi, 2010; Wang et al., 2014). Moreover, the maturation rate of oocytes, parthenogenetic activation rate of blastocyst rate are improved after melatonin treatment (Shi et al., 2009; Kang et al., 2009). Therefore, melatonin is beneficial for the oocytes maturation and embryonic development.

The androgenetic embryos had a poor developmental competence compared with the parthenogenetic embryos from morula to blastocyst stage. However, the androgenetic embryos had a higher cleavage rate than parthenogenetic embryos. Regulation of transcriptional activity is an essential role of paternal chromatin during early embryogenesis, and immediate transcriptional activity is associated with the pateral but not with the maternal chromatin, following sperm entry into the oocyte (Bui et al., 2011). Aoki, Worrad & Schultz (1997) reporte that one-cell mouse embryos have higher transcriptional activity in the male than that in female pronucleus. The histone H4 acetylation is probably associated with more effective commencement of DNA replication in the male pronucleus and with delay in completing DNA replication (Van Thuan et al., 2009; Bui et al., 2011). The differences between male and female genomes seem to arise from the maintenance of different levels of transcriptional activity in the female and male pronuclei (Bui et al., 2011). Therefore, the differential cleavage rate was related to significant genetic differences between androgenetic and parthenogenetic embryos.

Melatonin is a direct free radical scavenger and it acts as an indirect antioxidant by stimulating the expression of antioxidant enzymes (Manchester et al., 2015; Reiter et al., 2016). In the present study, a low expression level of ROS was detected in the parthenogenetic and androgenetic embryos after melatonin treatment. The result was consistent with the previous reports in porcine parthenogenetic embryos (Shi et al., 2009; Kang et al., 2009) and bovine IVF embryonic development (Tian et al., 2014; Wang et al., 2014). The relatively high oxygen stress cause the low efficiency of *in vitro* embryo production (Luvoni, Keskintepe & Brackett, 1996). The ROS production cause developmental arrests in mammalian embryos, embryonic fragmentation, or programmed cell death (Jurisicova, Varmuza & Casper, 1996; Yang et al., 1998). The ROS-induced programmed cell death results in a change of the mitochondrial function in embryos (Liu, Trimarchi & Keefe, 2000). Excessive production of ROS leads to an increasing risk of poor oocyte quality and developmental blocks in embryos (Tamura et al., 2008; Ishizuka et al., 2000). Perhaps it was one of the reason that melatonin improved parthenogenetic and androgenetic embryonic development and oocyte maturation via scavenging the ROS and anti-apoptotic. It has been reported that melatonin could decrease the level of ROS through its scavenging actions in the culture medium and improve *in vitro* maturation rate of oocytes and embryonic development in mice (Ishizuka et al., 2000), cattle (El-Raey et al., 2011) and pigs (Choi et al., 2008; Kang et al., 2009). The exact mechanisms underlying melatonin involvement in promoting the parthenogenetic and androgenetic embryonic development need to be investigated in future studies.

Melatonin regulates the ovarian function via hypothalamic-pituitary-gonadal axis in animal species (Tamura et al., 2009; El-Raey et al., 2011), and melatonin directly affects on ovary function. Some studies showed that melatonin was present in follicular fluid (Brzezinski et al., 1987), and melatonin receptors MT1 and MT2 are expressed in ovary. Melatonin in follicular improves the growth and maturation of COCs (Zhao et al., 2016; Kang et al., 2009) and induces the secretion of progesterone and estrogen in granulosa cells and luteal cells (Wang et al., 2012a; Battista, Rexroad Jr & Condon, 1987). Estradiol and progesterone are essential for follicular development and oocyte maturation (Okamoto et al., 2016). Progesterone enhances functional changes of cumulus cells and progresses meiotic maturation of oocytes during *in vitro* maturation of porcine COCs (Yamashita et al., 2010). In addition, the progesterone shows the ability of inhibiting the apoptosis in ovarian (Besnard, Horne & Whitehead, 2001) and COCs (O’Shea, Hensey & Fair, 2013). Estradiol could induce granulose cella and cumulus cell functional changes (Couse et al., 2005). Stepniak & Karbownik-Lewinska (2016) found that under physiological conditions estradiol contributed to protecting the ovary against oxidative damage to membrane lipids and nuclear DNA in porcine ovary. Coinciding with the previous studies, the present study indicated that melatonin stimulated the secretion of progesterone and estradiol in COCs, and the progesterone and estradiol production was higher in the 30 pmol mL^−1^ melatonin-treated group. Moreover, promoting the secretion of progesterone and estradiol might be due to enhancing the expression of *CYP11A1* and *CYP19A1* caused by melatonin treatment, which are responsible for regulating the biosynthesis of progesterone and estradiol in the ovary, respectively (Tamura et al., 2009; Pan et al., 2012). Furthermore, in response to higher hormone synthesis and higher level of *CYP11A1* and *CYP19A1*, StAR had a upregulating expression pattern response to melatonin treatment, which catalyzes translocation of cholesterol from relatively sterol-rich outer mitochondrial membrane to the relatively cholesterol-poor inner mitochondrial membrane, thereby initiating steroidgenesis (Pan et al., 2012; Miller, 2007; Christenson, Stouffer & Strauss 3rd, 2001; Zhen et al., 2014). Therefore, melatonin mediated estradiol and progesterone secretion through regulating the expression of steroidogenic genes (*CYP11A1*, *CYP19A1* and *StAR*). In addition, melatonin induced the secretion of progesterone and estradiol that was involved in improving the oocyte maturation.

The effects of melatonin on various physiological functions could be mediated by the receptors (MT1 and MT2) (Tamura et al., 2009). The current study showed that MT1 and MT2 proteins were expressed in bovine early parthenogenetic and androgenetic embryos. The presence of melatonin receptors in COCs and embryos indicate the potentially important role of melatonin in regulating oocyte maturation and embryonic development (El-Raey et al., 2011). In our previous study, the expressions of MT1 and MT2 were determined in bovine granulosa cells (Wang et al., 2012a). Kang et al. (2009) reported that the expression of MT1 gene was limited to cumulus and granulosa cells but not oocytes. El-Raey et al. (2011) revealed that MT1 gene was expressed in COCs, while MT2 gene was expressed only in oocytes. However, the melatonin receptor genes MT1 and MT2 are expressed in oocytes, cumulus cells, granulosa cells, and embryos (Tian et al., 2014; Wang et al., 2014). The mechanistic studies show that the beneficial effects of melatonin on bovine oocyte maturation are mediated via melatonin membrane receptors as the melatonin receptor agonist (IIK7) promotes this effect while the melatonin receptor antagonist (luzindole) blocks this action (Tian et al., 2014). Therefore, the presence of melatonin receptors in bovine early parthenogenetic and androgenetic embryos indicated the potentially role of melatonin in regulating parthenogenetic and androgenetic embryos development. However, the specific mechanisms that melatonin promotes bovine parthenogenetic and androgenetic embryonic development through the melatonin receptors require more intensive research.

Based on the present study, it provides further evidence that melatonin enhances bovine oocyte maturation and improves parthenogenetic and androgenetic embryonic development. Melatonin induced the secretion of progesterone and estradiol that was involved in improving the oocyte maturation. Moreover, melatonin regulated estradiol and progesterone secretion through mediating the expression of steroidogenic genes (*CYP11A1*, *CYP19A1* and *StAR*). In addition, reduction of ROS could be one of the mechanisms by which melatonin exerted its beneficial effects during and embryonic development. Therefore, the beneficial effects of melatonin on oocyte maturation and embryonic development could be used to enhance the efficiency of *in vitro* developed embryos.

##  Supplemental Information

10.7717/peerj.3485/supp-1 Supplemental Information 1 Effect of melatonin on *in-vitro* maturation of oocytes and the development of parthenogentic and androgenetic embryos. Raw data for hormone concentrations, maturation rate of oocytes, the development of parthenogenetic and androgenetic embryos, ROS of androgenetic and androgenetic embryos and the expression of steroidogenesis related genes (*CYP11A1, CYP19A1* and *StAR*).

10.7717/peerj.3485/supp-2 Supplemental Information 2 Proteins of β-actin in the morula stage of parthenogenetic and androgenetic embryos detected by the Western blotting method. 1, parthenogenetic embryos; 2, androgenetic embryos.

10.7717/peerj.3485/supp-3 Supplemental Information 3 Proteins of MT1 in the morula stage of parthenogenetic and androgenetic embryos detected by the Western blotting method. 1, parthenogenetic embryos; 2, androgenetic embryos.

10.7717/peerj.3485/supp-4 Supplemental Information 4 Proteins of MT2 in the morula stage of parthenogenetic and androgenetic embryos detected by the Western blotting method. 1, parthenogenetic embryos; 2, androgenetic embryos.

## Additional Information and Declarations

### Data Availability

The following information was supplied regarding data availability: Yang, Liguo (2017): Supplemental Dataset. figshare.
https://doi.org/10.6084/m9.figshare.4653460.v1.
