# Supplementary figures and images for: The effects of melatonin on bovine uniparental embryos development in vitro and the hormone secretion of COCs
Source: PeerJ. 2017 Jul 7;5:e3485. doi: 10.7717/peerj.3485 (PMC5502088; doi:10.7717/peerj.3485)

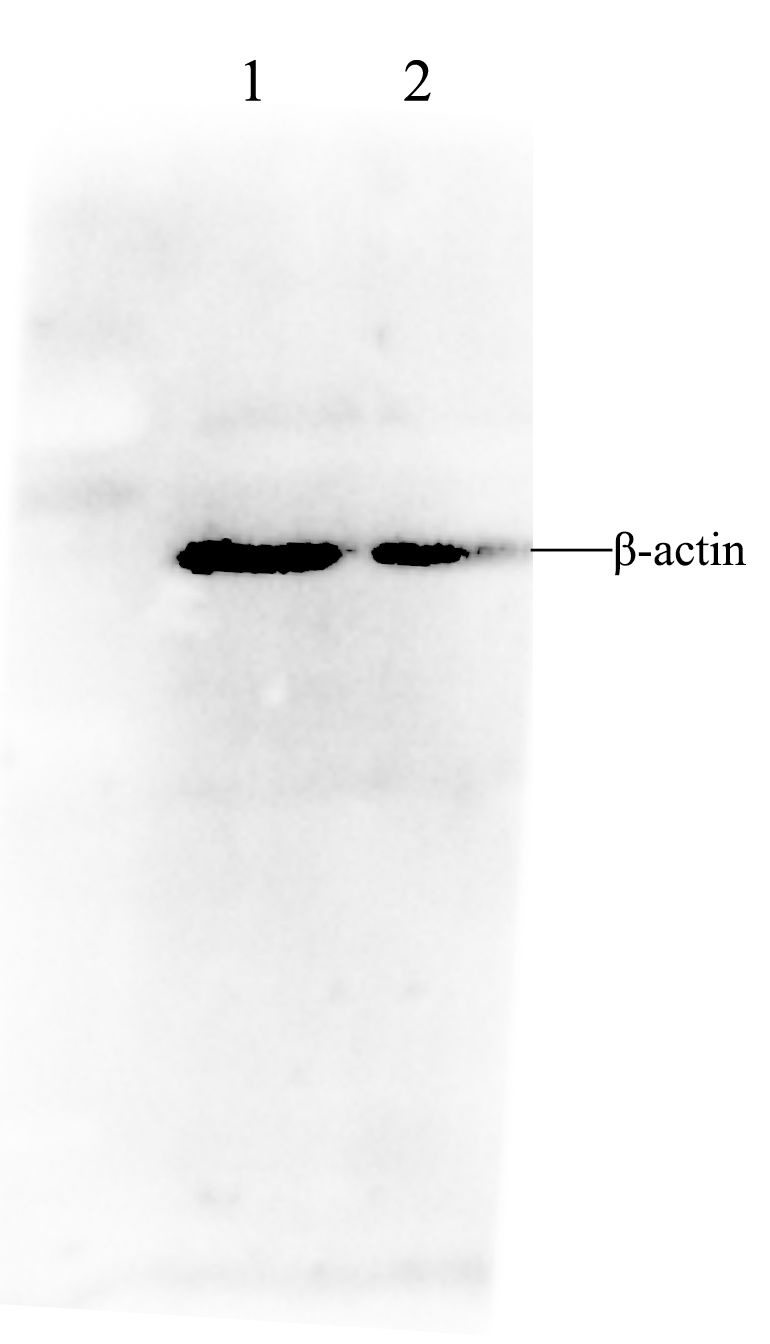

Supplement: Supplemental Information 2 — 1, parthenogenetic embryos; 2, androgenetic embryos. [file peerj-05-3485-s002.png]

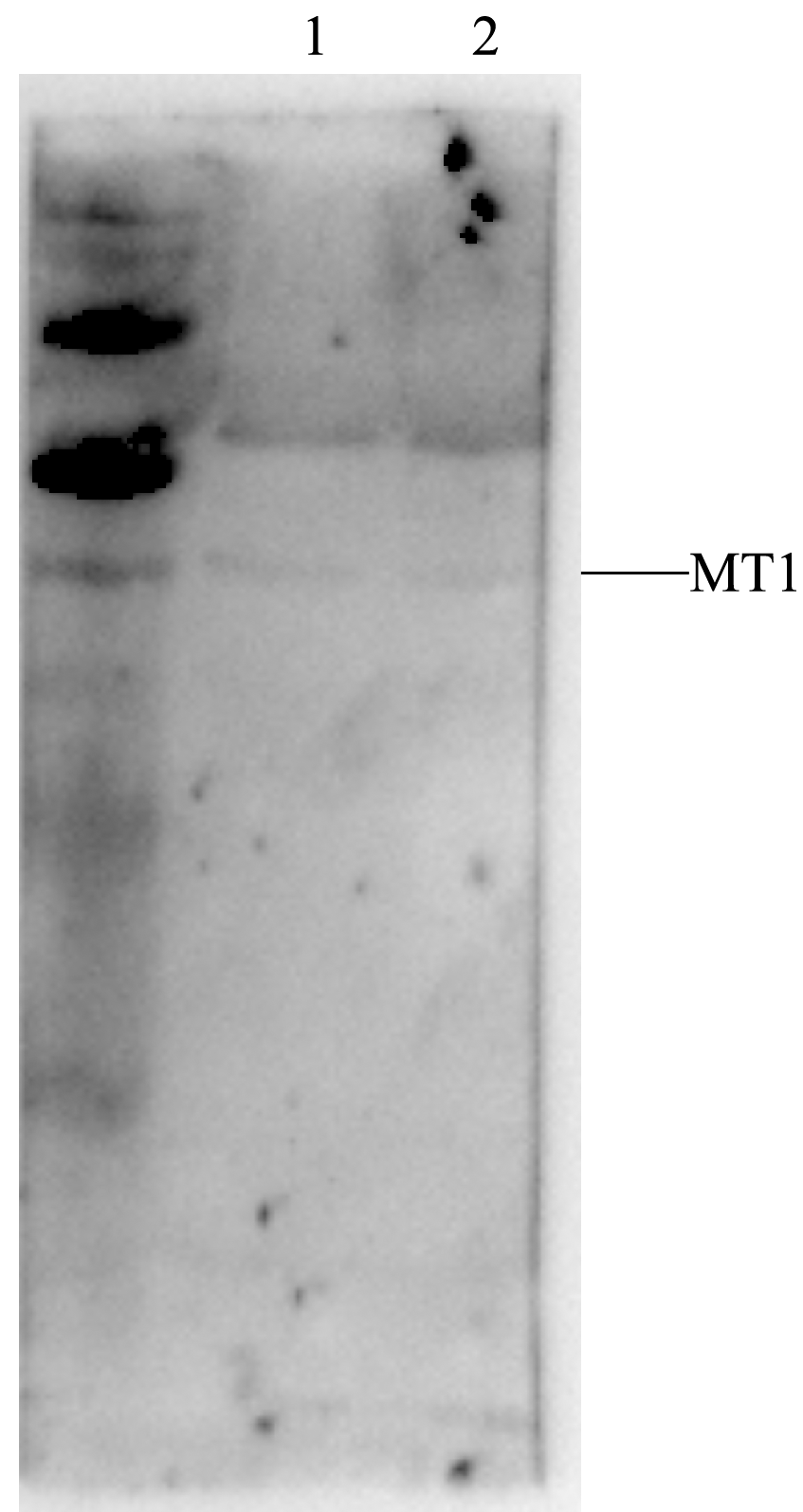

Supplement: Supplemental Information 3 — 1, parthenogenetic embryos; 2, androgenetic embryos. [file peerj-05-3485-s003.png]

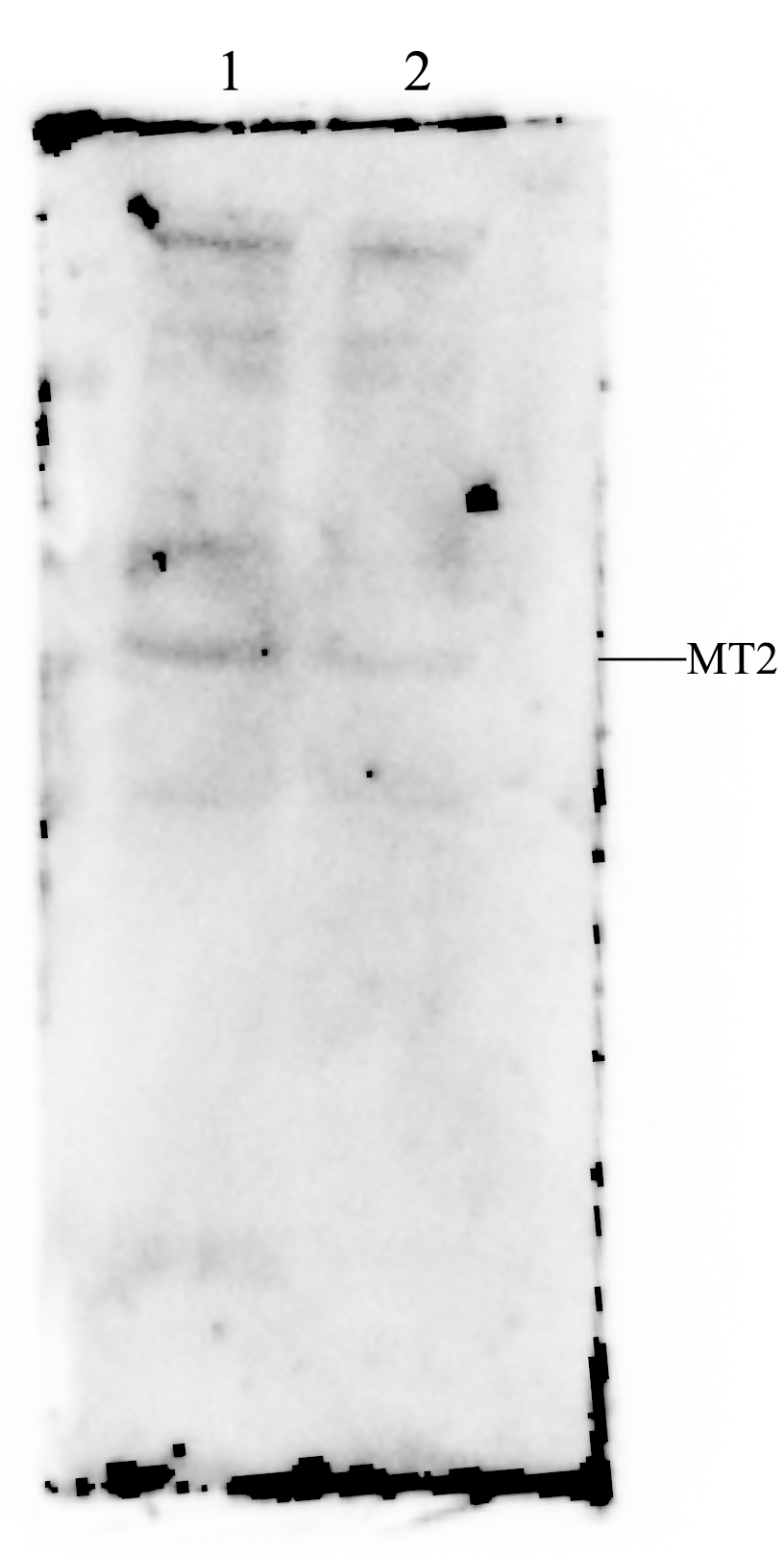

Supplement: Supplemental Information 4 — 1, parthenogenetic embryos; 2, androgenetic embryos. [file peerj-05-3485-s004.png]
